# COAST Development Group's international consensus guidelines for the treatment of canine osteoarthritis

**DOI:** 10.3389/fvets.2023.1137888

**Published:** 2023-08-03

**Authors:** Thibaut Cachon, Ole Frykman, John F. Innes, B. Duncan X. Lascelles, Masahiro Okumura, Pedro Sousa, Francesco Staffieri, Paulo V. Steagall, Bernadette Van Ryssen

**Affiliations:** ^1^Service de chirurgie, Campus Vétérinaire de Lyon VetAgro-Sup, Marcy l'Etoile, France; ^2^Unité de recherche ICE, UPSP 2007-03-135, VetAgro-Sup Campus Vétérinaire, Université de Lyon, Marcy l'Etoile, France; ^3^Herrgårdskliniken, Aneby, Sweden; ^4^Movement Veterinary Referrals, Runcorn, United Kingdom; ^5^Translational Research in Pain (TRiP) Program, College of Veterinary Medicine, North Carolina State University, Raleigh, NC, United States; ^6^Comparative Pain Research and Education Centre, Department of Clinical Sciences, College of Veterinary Medicine, North Carolina State University, Raleigh, NC, United States; ^7^Thurston Arthritis Centre, UNC School of Medicine, Chapel Hill, NC, United States; ^8^Center for Translational Pain Research, Department of Anesthesiology, Duke University, Durham, NC, United States; ^9^Department of Veterinary Clinical Sciences, Faculty of Veterinary Medicine, Hokkaido University, Sapporo, Hokkaido, Japan; ^10^Hospital Veterinari Montjuic - Vetpartners España, Barcelona, Spain; ^11^Section of Veterinary Clinicas and Animal Production, Department of Emergency and Organ Transplantations (D.E.O.T.), ‘Aldo Moro' University of Bari, Bari, Italy; ^12^Department of Clinical Sciences, Faculty of Veterinary Medicine, Université de Montréal, Saint Hyacinthe, QC, Canada; ^13^Department of Veterinary Clinical Sciences, Centre for Animal Health and Welfare, Jockey Club College of Veterinary Medicine and Life Sciences, City University of Hong Kong, Hong Kong, Hong Kong SAR, China; ^14^Department of Medical Imaging and Small Animal Orthopedics, Faculty of Veterinary Medicine, Ghent University, Ghent, Belgium

**Keywords:** dog, pain, osteoarthritis, staging, management, treatment guidelines, COASTeR, COAST

## Abstract

This report describes consensus guidelines and recommendations for the treatment of canine osteoarthritis (OA) according to the “Canine OsteoArthritis Staging Tool excluding radiography” (COASTeR) stage of OA, by the COAST Development Group. The recommendations are based on evidence-based medicine and clinical experience and are proposed with international relevance in mind. The aim is to provide veterinarians with a practical reference to consolidated information and to support the development of patient-specific OA management protocols and informed treatment choices based on the stage of OA.

## Introduction

Osteoarthritis (OA) is a progressive, degenerative disorder of synovial joints (1). It is characterized by pain and low-grade chronic inflammation, with structural and functional deterioration of the joint (2–5). In dogs, it is commonly initiated early in life by developmental joint disease (e.g., hip dysplasia) (6, 7). Joint trauma is also another important OA initiator in this species (6, 7). Diet, obesity, genetics, age, breed, and environment are risk factors that can influence OA development and progression (7–9). Currently, the disease itself is incurable, and patient care is primarily focused on minimizing the negative consequences related to pain, mobility impairment, and decreased quality of life (10). In addition, the need to consider the potential sustained negative effects of pain, central sensitization, and activity impairment on the affective state (anxiety, depression, and sleep impairment) and cognitive dysfunction of dogs is increasingly understood (11–15).

Unfortunately, the complex etiology, multi-factorial influences, large individual-to-individual variations in disease burden and impact, and the chronic, progressive nature of the disease make canine OA management particularly challenging. These challenges are compounded by patient-specific medical requirements, individual variability in response to treatment, pet caregiver considerations, and the time constraint of many first-opinion evaluations. Geographical differences in products approved for veterinary use, plus variability in the level of adoption, experience, and comfort with certain therapeutic strategies, also influence treatment choice. Although a multi-modal plan, incorporating both pharmacological and non-pharmacological treatment modalities, is a well-accepted approach in OA management, there can be notable differences between the treatments and the duration of product administration recommended by veterinarians. An evidence-based, standardized approach to treatment and consensus general management of dogs with OA may help to further optimize patient care.

In 2018, the COAST Development Group proposed the Canine OsteoArthritis Staging Tool (COAST) as a system to define the stages of canine osteoarthritis (16). The main objectives of COAST were to promote a more standardized approach to diagnosis and monitoring of OA, increase opportunities for pet caregiver education, make earlier detection of OA a possibility, and support the evaluation of response to therapy. In a subsequent observational study evaluating the clinical application of COAST, the staging tool was shown to be suitable and valid for the assessment of the severity of osteoarthritis in dogs and correlated well with clinical opinion (17). Staging of disease has been found to be an effective platform on which to base treatment recommendations, and this approach is well-established in other fields of veterinary medicine such as cardiology (18, 19), nephrology (20), dermatology (21), and oncology (22).

The objective of this COAST Development Group consensus guidelines is to provide a practical, expert, and evidence-based, COAST-stage-led approach to the treatment of OA, and the recommendations have been proposed with international relevance in mind. Given radiographic OA changes do not correlate well with clinical function and pain (23, 24), and the recommendations are presented according to the COASTeR stage (COAST excluding Radiography from the stage calculation). The foundational elements of the consensus constitute management approaches applicable to most, if not all, patients. Additional treatment options are then recommended in a stepwise, evidence-, and expert opinion-based manner, for clinician consideration according to disease severity and individual patient requirements. The COAST Development Group intends to encourage a more consistent approach to building canine OA management protocols but to leave enough flexibility for the management of patients with differing needs and treatment outcomes. Overall, the guide is intended to provide clinicians with a reference of consolidated information and to support benefit: risk evaluations and informed treatment choices when building tailored treatment plans for dogs with OA.

## Background

### The COAST Development Group

The COAST Development Group is a geographically diverse group of nine international veterinarians actively working in the fields of small animal orthopedics, anesthesia, and pain research and management.

### The Canine OsteoArthritis Staging Tool

The Canine Osteoarthritis Staging Tool forms the diagnostic framework for the COAST canine OA treatment guidelines. This staging tool encourages the collection of information from the pet caregiver, the veterinarian, and the wider care team, to help determine the impact of OA on the dog's joints and general wellbeing. COAST-recommended evaluations include completion of a validated pet owner/caregiver questionnaire (clinical metrology instrument or client-reported outcomes measure), recording the pet caregiver's opinion of the level of the dog's pain, and a full observational and hands-on orthopedic examination (posture, motion, and physical examination of the joints) and radiography (16). The information is combined to provide the overall COAST stage of OA (Table 1).

**Table 1:** The COAST stages of osteoarthritis (OA) and their corresponding descriptors.

**COAST stage**	**Description**	
0	Pre-clinical	Clinically normal. No OA risk factors
1			Clinically normal but OA risk factors present
2	Clinical	Clinical signs of mild OA
3			Clinical signs of moderate OA
4			Clinical signs of severe OA

Radiography is important for confirmation of the diagnosis of the OA disease, the re-assessment of joints in complex and/or deteriorating cases, and the overall staging of canine OA. However, it is generally accepted that the treatment of OA should be based on clinical rather than radiographic signs. The COAST Development Group treatment recommendations are therefore presented by the COAST stage determined after radiography is excluded (COASTeR stage).

### Determining the COAST Development Group recommendations

The COAST Development Group consensus recommendations for the treatment of dogs with OA were primarily defined by the availability and quality of evidence for each treatment modality. However, although numerous drug and non-drug options are frequently incorporated into multimodal management protocols for canine OA, relatively few of them have been extensively evaluated and relatively few drugs/biologics are approved for use in dogs. Substantial evidence of efficacy in this species was therefore found to be lacking for many OA treatment options. Individual interpretation of any data available, an evaluation of the benefit vs. harm for each intervention, and any personal experience insights were also included in the decision-making. Voting for surgical procedure recommendations was limited to the orthopedic surgeons in the group. The geographical diversity of the group ensured that the team considered a wide range of local or regional approaches to veterinary medicine, and, in some cases, differences in product approval or technique adoption did influence the overall strength of the group recommendation for a particular treatment. It is envisioned that country- or region-specific treatment recommendations for dogs with OA will complement the international treatment guidelines, by providing more detail about geographically specific indications for use, management approaches, and opinions. Treatment decisions were based on the most scientifically robust, up-to-date information available at the time.

### Incorporation of available evidence

A literature search, incorporating the MeSH terms “canine or dog” and/or “osteoarthritis” and various treatment options (depending on the subject being explored), was conducted using the search engines PubMed, Ovid, and Google Scholar. Each member of the COAST Development Group factored the levels of published evidence (Table 2) into their voting on recommendations and classification (strength) of treatment recommendations (Table 3).

**Table 2:** Definitions of levels of evidence used by the COAST Development Group for the treatment recommendations based on the available literature.

**Levels of evidence**	
High	Randomized controlled trials in dogs
	Prospective, non-randomized controlled trials in dogs (adequate sample size/ no major methodological flaws)
Medium	Experimental laboratory trials in dogs
	Retrospective clinical studies with intervention and control groups in dogs
Low	Case series or case reports in dogs without control groups
	Studies in other species
	Expert opinion

Adapted from (25).

**Table 3:** General guide to interpreting the authors' levels of recommendation based on voting.

**Level of recommendation**	**General interpretation of this recommendation**
*Unanimous recommendation*	•Unanimous support for this management approach/treatment for dogs with the specified stage of OA. •Sufficient high-quality evidence, supported by personal experience. •The benefit: harm evaluation favors treatment use.
*Majority recommendation*	•No unanimous support for this management approach/treatment for dogs with the specified stage of OA but sufficient evidence for most of the group to recommend it as a treatment option. •The benefit: harm evaluation favors use in the opinion of the majority.
Minority recommendation	•No unanimous support for this management approach/treatment for dogs with the specified stage of OA. •Weaker evidence-based support relative to other options, with only a minority of the group recommending it as an option. •The benefit is considered at least equal to or better than the risk. •Individual patient factors are likely to be a significant consideration when considering the use.
Not a recommendation currently	•Unanimous agreement that this approach could not currently be recommended. •Insufficient evidence and lack of personal endorsement. •In some instances, the benefit: harm evaluation was of concern.

Each author voted independently, based on their understanding of, and interpretation of, the literature (e.g., Table 2) and their personal experience. Thus, levels of recommendation are based on the sum of individual authors' interpretations and voting, not a group consensus on a set of criteria.

### OA management and treatment modalities evaluated

The COAST Development Group focused its initial evaluations on established OA management approaches, in use in more than one country, to ensure a reasonable balance of published data and clinical experience. Approximately 40 surgical, pharmacological, biologic, and non-pharmacological OA treatment and management options were included (Appendix 1).

COAST Group recommendations for physical and machinery-applied techniques to optimize physical function are provided under the umbrella term “rehabilitation/physical therapy,” rather than each approach being listed individually. Although not all the treatment modalities within this specialist field necessarily have the same level of evidence to support use, the COAST Development Group recognized the need to encourage consultation with certified physical therapists or rehabilitation specialists and for OA management protocols to be developed flexibly, according to the requirements of the individual patient. Professional recommendations may differ depending on the levels of expertise, experience, and availability of equipment. Individual patients may also respond more positively to certain techniques, and pet caregiver opinion or limitations may influence treatment choice. The treatment modalities considered by the COAST Development Group under the term “rehabilitation/physical therapy” are listed in Table 4.

**Table 4:** Physical rehabilitation treatment modalities that were considered by the COAST Development Group are included under the term “rehabilitation/physical therapy.”

**Professional administered or supervised techniques**			**At-home treatment modalities (Only after professional consultation)**
**Manual therapy**	**Movement and exercise**	**Machinery or instrument applied**	**Manual therapy or exercise**
Cryotherapy/thermotherapy	Hydrotherapy	Acupuncture/electroacupuncture	Hot/cold therapy
Massage	Proprioceptive exercise	Electrical nerve stimulation	Massage
Myofascial release/trigger point therapy	Therapeutic exercise	Extracorporeal shockwave therapy	Passive range of motion
Range of motion (assisted)	Treadmill	Photobiomodulation	Therapeutic exercise
Range of motion (passive)		Pulsed electromagnetic field therapy	
Traction		Ultrasound	

Listed in alphabetical order.

Following the draft development of the COAST Group treatment recommendations, a new therapeutic class (a canine monoclonal antibody targeting Nerve Growth Factor) was introduced for dogs with osteoarthritis, prompting a supplementary evaluation of treatment options and an update to the draft consensus (June 2022). Relative to other treatment options evaluated, more complex user scenarios could not be clearly defined due to the finite amount of data available (as for any new product) as well as the clinical experience being limited by geographic availability and the product's relatively short time in the market. As a result, the current COASTeR stage-specific treatment recommendations for the anti-nerve growth factor monoclonal antibody simply reflect the approved indications for use. Although this approach will leave veterinarians with questions regarding how to effectively integrate this newer treatment option into more established OA management protocols, the COAST Development Group agreed that, based on the evidence currently available, at an international level, it was inappropriate to differentiate and define when to use, or when not use, different classes of approved products with the same or a similar indication for use. During the evaluation discussions, the COAST Group did note factors that they would personally consider before the selection of NSAIDs or an anti-NGF mAb and have provided an overview to support informed treatment choice and responsible product use (Appendix 2).

The absence of any specific OA management approach from the consensus is not a negative opinion of product or treatment modality use unless specifically stated in the guidelines. It only indicates that the product was not evaluated on this occasion. Updates to the COAST Development Group consensus are envisaged for the future, and additional treatment options will likely be incorporated.

## An overview of the consensus recommendation approach

**Figure 1:**
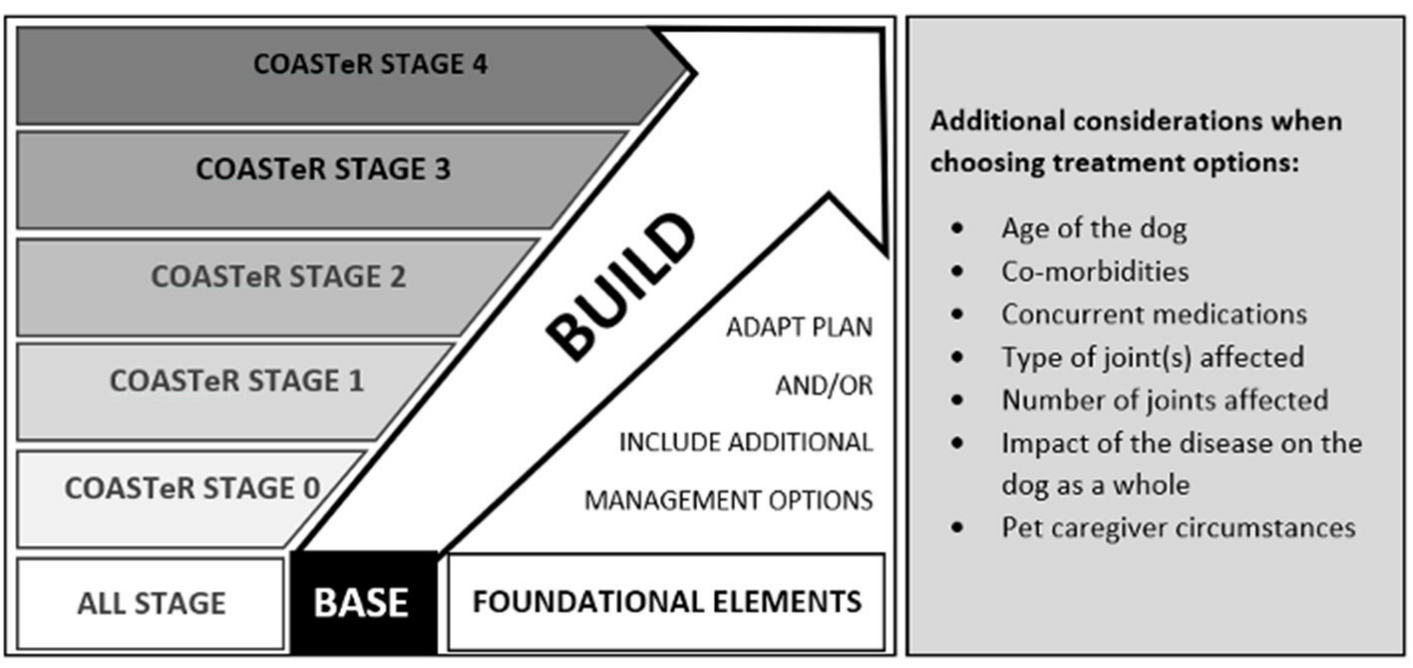
Simple and practical recommendations of the COAST Development Group using a “base and build” approach for every canine OA management protocol.

The COAST Development Group recommends a simple but practical “base and build” approach for individual canine OA management protocols (Figure 1; Table 5), using the “COAST Stage excluding radiography” (COASTeR stage) to guide treatment and management choice.

**Table 5:** A summary of the steps involved in the practical implementation of the COASTeR consensus guidelines for the treatment of canine osteoarthritis.

**Step**		**Action**	**Details**
1	Base	Initiate foundational elements	•Applicable to all stages of OA. •Pro-actively seek opportunities for OA evaluation and education according to patient criteria such as life stage.
2	Stage	Determine the COAST Stage of OA (overall) and COASTeR Stage (to guide treatment)	•Include radiography when determining the overall COAST Stage, but exclude radiography when determining the COASTeR Stage that guides treatment. •Identify patient and/or pet caregiver factors that may affect treatment choice.
3	Build	Expand foundational elements according to COASTeR Stage	•Address or introduce specific topics and items to further develop foundational elements according to the stage of the disease e.g., a weight management program for COASTeR Stage 1 overweight or obese dogs.
4	Build	Introduce COASTeR Stage-specific treatment or management options	•Develop the management plan by incorporating other COASTeR stage appropriate treatment/management options. •Consider the strength of the COAST Development Group recommendation during treatment selection. •Incorporate specific patient and/or pet caregiver requirements as appropriate.
5	Assess	Evaluate treatment response	•Continue to monitor the patient at appropriate intervals.
6	Build	Add/adjust treatment or management options according to response or disease progression	•If necessary, adjust the treatment plan due to a positive outcome or because further improvement is required. •Consider the strength of the COAST Development Group recommendation during treatment selection. Progression of OA to the next COASTeR Stage will require a more extensive re-evaluation of the management plan. Some treatments are more strongly supported, or different treatments are recommended, for dogs with more severe diseases. •Incorporate specific patient and/or pet caregiver requirements as appropriate.
	Repeat	Regular assessment of the patient with protocol adjustments if required

- The foundational elements form the “base” of the recommendations.
- Education and proactive evaluation of lifestyle factors constitute most of the foundational elements and are therefore applicable to all patients, including those without clinical signs of osteoarthritis. They are presented according to life-stage or educational opportunity.
- COASTeR stage-specific management and treatment recommendations make up the “build” elements. These elements are intended to expand or build on the foundational elements, in a stepwise butflexible manner.

° For each build item that is not applicable to the patient (e.g., the dog is already of optimal body weight, so a weight management program is not required), it is recommended to refer to COASTeR Stage 0 where advice for caregivers of dogs without OA risk factors is provided.
° For each build item that is applicable to the patient, the group suggests building on the previous stage discussions and expanding the topics according to the current stage of OA.

The objective is to develop an OA management program using a consistent approach but with enough freedom of choice to meet the requirements of each patient and pet caregiver.

### Foundational elements: recommendations applicable to all stages

The COAST Development Group recommends implementation of the foundational elements as the base of the consensus for all dogs, regardless of OA status, unless there is a patient or pet caregiver's specific reason why doing so is not appropriate. For dogs at risk of OA, the foundational elements can help to increase pet caregiver awareness and education, are important for the mitigation of OA risk factors, and can help maintain or improve the dog's strength and fitness, as well as facilitate a timely OA diagnosis. For dogs with OA, these non-drug/non-surgical options form a solid base on which to build the COASTeR stage-specific OA management protocols. The objectives of the foundational approach will differ depending on the age of the dog and other factors, including the experience and commitment of pet caregivers, and treatment option availability. Summary tables of the recommendations are provided according to life stage and specific education area opportunities (Tables 6, 7).

**Table 6:** Evaluation opportunities applicable for “all-stages” (COASTeR Stage 0–4) and listed according to life stage, the definition of which will vary with breed life expectancy (26).

**Life stage**	**Evaluation opportunities**	**Objectives**
Puppy	Health check and first vaccinations	•Introduce COAST/Familiarize pet caregivers with the concept/terminology •Begin pet caregiver/ pet owner disease education about OA (reduce risk factors) •De-sexing discussion (positives, negatives, and timing) •Identify any individuals that could benefit from preventive surgery
Young adult	Health check	•Assess COAST/COASTeR Stage to determine an individual baseline and open discussion about OA (e.g., at-risk breeds) •Dietary/nutrition discussions •Growth rate monitoring •Body weight monitoring •Determining body and muscle condition score •Exercise plan •Ongoing pet caregiver education •Pre-neuter discussion (if still applicable)
Mature adult	OA education programs	•Focused OA education program for pet caregivers (continue engagement and encouragement) •Assess COAST/COASTeR Stage (for adult dog baseline or if any changes/cause for concern)
	Annual health check	•Evaluation for clinical signs of OA •Ongoing pet caregiver education (brief) •Recommend a follow-up COAST/COASTeR evaluation if necessary
Senior	Senior/geriatric wellness	•Increase the frequency of OA evaluation visits for senior dogs. Consider breed size and average life expectancy (i.e., dogs entering the last 25% of their estimated lifespan). •Conduct a COAST/COASTeR evaluation or re-evaluation
All ages	Opportune	•Quick evaluation for clinical signs of OA •Ongoing pet caregiver education (brief) •Recommend a follow-up COAST/COASTeR evaluation if necessary

**Table 7:** Education opportunities and their objectives that are applicable for all canine life stages (COASTeR Stage 0–4).

**Education opportunities**	**objective**
Pet caregiver	•Importance of OA assessments in young dogs e.g., Restricted window of opportunity for preventive surgery •Common causes of OA Developmental joint disease and joint trauma •Osteoarthritis disease awareness: A progressive disease of the joints that can affect dogs of different sizes, breeds, and ages •Basic understanding of risk factors and how to avoid them e.g., overweight/obesity •Importance of regular assessments/check-ups, both in-clinic and watching out for changes at home •Discuss other foundational education items (below)
Weight optimization	•Measure body weight and evaluate relative to body condition score, muscle condition score, and age •Discuss the importance of maintaining or achieving an optimal body weight
Nutrition/dietary discussion	•Diets balanced for life stage •Energy and protein requirements according to breed/size •Puppies: Caloric intake appropriate to rate of growth •Puppies: Nutrient deficiency OR excess can contribute to the development of OA Calcium: Phosphorus imbalance (usually calcium deficiency with a relative phosphorus excess) can lead to joint incongruity. Calcium excess can lead to severe disturbances in skeletal development, growth, and mineralization •Emphasize benefits of overall calorie restriction/ lifelong maintenance of optimal body condition score
Appropriate exercise	•Emphasize the benefits of exercise to musculoskeletal and general health •Important for musculoskeletal strength (core and joints) •Age and breed appropriate •Regularity is required: - Daily exercise - Consistency in duration (but may change according to age/life stage) •Avoid/minimize high-impact activities, sudden loading, or excessive exercise, unless the dog is conditioned (training gradually resulting in physical fitness) to perform this.
Rehabilitation/physical therapy	•Professional guidance (appropriate exercise program), ideally with a certified physiotherapist •Specific exercises to support balance, strength/tone, endurance, and flexibility •Injury prevention strategies •Mental stimulation •Easy, every day at-home exercise (dog health advocacy for pet caregivers)

The COAST Development Group did not discuss prophylactic treatments against infective or parasitic-based joint disease, but all agree that such approaches are appropriate, and recommendations will vary based on geographical location. Such recommendations should be incorporated into local OA prevention and treatment guidelines.

### Selecting COASTeR stage-appropriate treatment options

The COASTeR Stage of OA is required for the continued build and optimization of OA management protocols according to the COAST Development Group treatment recommendations. Patients have changing and generally expanding needs for treatment as disease severity increases, and the list of options and the strength of group recommendation may differ depending on the COASTeR Stage of OA. For each stage, the COAST Development Group advises selection from the treatment options with the strongest evidence (unanimous recommendation) first. If the patient requires additional support, treatment options with a lower strength of evidence can then be considered. Treatment modalities with majority or minority recommendation in one COASTeR Stage may be more strongly recommended in the next COASTeR stage. Due to the possibility of utilizing options with a lower strength of evidence when needed, dogs receiving a long-term management plan and advancing to the next COASTeR Stage of OA may already be receiving the treatments unanimously recommended for that new stage. This situation necessitates the immediate consideration of options with weaker evidence within that new stage. Similarly, complex or challenging cases are likely to require treatment selection from a more expansive list of recommendations, even if evidence for use is not as strong. The COAST group consensus also supports the adaptation of previous protocols (e.g., a dosing increase or decrease), according to the patient's needs. Products approved for use in dogs with OA tend to have the most robust data supporting efficacy and safety, so the COAST approach aligns with other risk-based decision approaches that allow the use of clinical judgment when authorized products are not available. Individual product selection within this overarching approach is often influenced by clinician preference, familiarity, and individual patient and/or pet caregiver requirements.

Where appropriate, the COAST Development Group suggests a minimum duration of use for some treatment options, plus recommended re-evaluation intervals. The use of products at an efficacious dose for a clinically appropriate duration of time is strongly encouraged, especially when optimizing the initial phase of treatment. This enables ongoing OA management protocols to be further developed based on the greatest functionality achievable by the patient at the time. Re-evaluation of the patient at regular intervals is important for confirming both efficacy and tolerability or for enabling the timely detection of treatment-induced adverse effects. Adjustment of the treatment plan following the initial management phase is expected and should be dependent on the response (positive or negative) to therapy. It must be remembered that few veterinary products have been evaluated with other medications, so reference to specific product label requirements and regular patient monitoring is strongly recommended when combining treatment options. Additionally, product labels may vary according to the country of origin.

### Teamwork

The COAST Development Group recommends utilizing a multi-disciplinary team approach whenever possible. OA management is complex and at a minimum involves significant time commitments for pet caregiver education and explanation of care plan updates. Veterinary technician/nurse-led appointments, as allowed within territorial regulatory frameworks, plus the inclusion of a wider array of treatment modalities provided by fellow animal healthcare professionals can help optimize multimodal management.

### Patient re-evaluation

The COAST Development Group considers patient re-evaluation to be a mandatory component of a successful canine OA management plan. As a minimum, patients should be re-evaluated:

- at regular “stage-appropriate” intervals
- following the introduction of new treatments
- with any reported deterioration.

The COAST Development Group endorses all forms of patient follow-up, including telehealth, but recommends using video of the dog in the home environment, or of the dog performing daily activities, as well as an in-person, hands-on evaluation at least two to three times a year.

### Referral

Referral to a pain control specialist and/or orthopedic surgeon, or blended specialist, with active interests in diagnosis and management of canine OA-associated pain for OA diagnosis and/or treatment is always an option for any dog. This is particularly important for the following patients:

- Young dogs or severely affected dogs, e.g., if surgery could be a consideration or if they are facing long-term, and potentially complex, medical management.
- Young dogs with growth disturbances.
- Deteriorating or otherwise difficult-to-manage cases with potential refractory pain and significant quality of life compromise.
- Before the end-of-life decision.

## Treatment recommendations

**COASTeR Stage 0:**

No clinical signs of OA. No OA risk factors were identified.

*Unanimous recommendations*: ^*^

FOUNDATIONAL ELEMENTS: ^*^9 out of 9

Initiate relevant “all-stage” foundational elements (Tables 6, 7), if not already actioned.

BUILD ELEMENTS

Expand discussions by introducing COASTeR stage 0 applicable topics:

- Non-Drug/Non-Surgical

Disease Education: ^*^9 out of 9

Continue to build pet caregiver understanding and engagement.

° Focus on disease education.
° Reinforce the need for continued pet caregiver diligence and ongoing control of known OA risk factors (minimize the risks of developing OA on a long-term basis).
° Recommend regular (life-stage dependent) appointments for patient monitoring and pet caregiver education, prioritizing convenience for the pet caregiver (e.g., combined with annual health checks).
° If possible, initiate or utilize vet technician/nurse-led appointments to provide more flexibility.
° Utilize engaging educational tools and resources whenever possible.
° Balance owner expectations. There is still a lot to be understood about the risk of OA for dogs that do not have obvious predisposing factors.

Nutrition/Diet: ^*^9 out of 9

Nutritional requirements will alter throughout life and dietary changes will be required. A specific joint diet/functional food is not currently recommended.

Exercise: ^*^9 out of 9

Exercise should be breed-and life-stage-appropriate and adapted as required.

Avoid high-impact exercises and activities that encourage sudden changes in direction (twists and turns) in unconditioned dogs. Consider injury/OA risk associated with these activities in conditioned dogs.

Physical therapy: ^*^9 out of 9

Physical therapy is considered optional for this stage of OA, but programs focused on building and enhancing exercise programs to minimize the risk of injury can be beneficial.

**COASTeR Stage 1**

No clinical signs of OA. One or more risk factors were identified.

Unanimousrecommendations^*^:

FOUNDATIONAL ELEMENTS: ^*^9 out of 9

Initiate “all-stage” foundational elements, if not already actioned.

BUILD ELEMENTS

Expand discussions by introducing COASTeR stage 1 applicable topics listed below. Refer to COASTeR Stage 0 recommendations if the listed item is not a risk factor for the individual patient.

- Non-Drug/Non-Surgical

Disease Education: ^*^9 out of 9

Continue to build pet caregiver understanding and engagement.

° Continue disease education, including awareness and understanding of disease progression.
° Focus on mitigating identified risk factor(s) whenever possible. Increase awareness of other OA risk factors and how to avoid them.
° Recommend a regular and frequent sequence of follow-up appointments. The frequency will be determined by risk mitigation objectives (e.g., weight management) and specific needs for disease monitoring and pet caregiver education.
° Integrate vet technician/nurse-led appointments and engage tools and resources whenever possible.

Body weight: ^*^9 out of 9

Discuss how excess body weight/obesity increases joint loading and has a metabolic contribution.

Emphasize the importance of achieving and maintaining an optimal body weight over the dog's lifetime.

Quality supportive materials and weight control information are available from other guideline committees (27, 28).

Nutrition/Diet: ^*^9 out of 9

Support the pet caregiver in sourcing a nutritionally balanced, breed, and life-stage appropriate diet for the dog.

Transition to a joint diet/functional food and/or introduction of nutritional supplements is not unanimously recommended (refer to *minority recommendations*), but awareness and understanding of these options enable pet caregivers to make an informed choice.

Modified Exercise: ^*^9 out of 9

Develop a breed/life-stage/life-style appropriate exercise program and/or refer to a physical therapist/rehabilitation specialist:

° Professional help to initiate an appropriate exercise program, or to further develop an established program.
° Encourage a full rehabilitation/joint support program if a joint injury/trauma (current or previous) has been identified.
° Minimizing deleterious effects on joints should be a focus.

Focused/more frequent patient evaluations: ^*^9 out of 9

Disease-focused and/or more frequent evaluations are recommended for the following dogs:

° Breeds with a genetic predisposition for OA, working and sporting dogs, dogs with a history of joint trauma, needing weight management, and/or entering senior life stage.
° Dogs diagnosed with developmental joint disorder(s). The first full evaluation should incorporate COAST staging, including radiography.

Dogs < 1 year of age
The joint(s) affected and geographical differences in approach are both factors affecting the frequency of evaluations and treatment/management recommendations. In general, additional examinations in the period between 3 months and 1 year of age (i.e., two or three visits during the dog's growth phase) are supported. The use of a radiographic screening program is encouraged and initiation of evaluations early enough in the dog's life should be ensured to allow for appropriate timing of surgical intervention (see surgical recommendations). There are multiple radiographic screening programs available, and selection is likely to be determined by which schemes are supported in individual countries.

Dogs > 1 year of age
Reassess the patient every 6 months.
Include the pet caregiver's completion of a validated clinical metrology instrument and a more extensive physical/orthopedic examination.
Additional radiographs are not required unless there is a major change (such as a notable increase in discomfort and/or a decrease in activity/mobility).

Majorityrecommendations^**^:

- Surgical (vote restricted to orthopedic surgeons only):

Referral to an orthopedic surgeon is always an option.

° Juvenile Pubic Symphysis (JPS): ^**^5 out of 6
Recommended as preventive surgery for dogs if the patient meets specific criteria (dogs < 5 months of age with joint laxity distraction indices (DIs) of 0.5 to 0.7).
° Other preventive surgeries, e.g., Double or Triple Pelvic Osteotomy can also be considered but have lesser strength of evidence than JPS.

Excellent functional outcomes are now reported for the majority of patients following total hip replacement (THR), so avoiding preventive surgery and performing a THR later in life if required, is another option.

Preventive or curative surgeries should be applied when necessary.

*Minority recommendations*: ^***^

- Non-Drug/Non-Surgical

Nutrition/diet:

These *minority recommendations* only apply to dogs at risk of developing OA that have reached adulthood.

□ Food
- Joint diet/functional food: ^***^ 4 out of 9

Consider introducing a joint diet/functional food, scientifically developed to provide a balanced ratio of omega-3:omega-6 fatty acids, derived from appropriate ingredients in sufficient quantities.

Factors affecting group decision:

The remainder of the group felt that a joint diet/functional food was not required if the dog's diet was nutritionally balanced. Obesity risk and the potential for additional expense were the main reasons why this item was limited to a minority recommendation.

□ Supplement
- Omega-3 fatty acid supplements:^***^ 1 out of 9.

Consider omega-3 fatty acid supplements as an alternative to a joint diet/functional food. A recommended dosage specifically for dogs at risk but with no clinical signs of OA is not available, although a combined EPA/DHA dose (mg) of 30^*^BW^0.75^, with BW measured in kilograms (kg), is the allowance reported by the National Research Council (NRC) for adult maintenance (29).

Factors affecting group decision:

Most of the group felt that nutritional supplements were not required at this stage if the dog's diet was nutritionally balanced. Additional cost, sourcing a good quality supplement without other unwanted ingredients, obesity risk, and ability to administer a sufficient dosage were the main factors driving this decision.

Essential fatty acids have the largest evidence base of nutritional supplements. Other supplements have not been listed for COASTeR Stage 1 dogs due to their more limited evidence base. Administration of other ongoing nutritional supplements may continue at the pet caregiver's request.

The potential additional benefits of functional food, such as overall calorie control and provision of other nutrients, were the reason why fewer COAST Development Group members supported nutritional supplements than joint diets for these dogs.

**COASTeR Stage 2**

Clinical signs of OA. Mild osteoarthritis pain.

Unanimousrecommendations^*^:

FOUNDATIONAL ELEMENTS: ^*^9 out of 9

Initiate “all-stage” foundational elements, if not already actioned.

BUILD ELEMENTS

Expand on COASTeR stage 0 or 1 discussions as appropriate by introducing COASTeR stage 2 applicable topics listed below.

- Non-Drug/Non-Surgical

Disease Education: ^*^9 out of 9

At this stage of OA, pet caregiver education is critical from the first visit. It is required for setting expectations and for ensuring a commitment to care.

° Ensure that pet caregivers understand that OA is a life-long, progressive disease that can be managed, but requires regular re-evaluations and commitment to a long-term care plan. Regular re-visits are important for patient monitoring, evaluation of response to treatment, and for strengthening pet caregiver engagement.
° Mitigate any identified OA risk/progression factor(s) whenever possible.
° If not already done, schedule a complete COAST staging evaluation and confirm OA by radiography. Educate about the benefits of radiography, e.g., determine if there are any underlying factors such as developmental joint disease (e.g., elbow dysplasia, hip dysplasia, and osteochondritis dissecans) and exclude other diseases.
° Include a COASTeR stage 2 treatment-appropriate schedule of visits (e.g., an average of two to four times a year): Set the expectation for relatively frequent re-evaluations following treatment plan initiation, with a reduction in revisits once optimal function is achieved.

▪ Flexibility in the number of visits may be required depending on the response to treatment and other factors.
▪ It is increasingly important to incorporate vet technician/nurse-led and other multi-disciplinary team appointments, plus educational tools.

° Blood work is recommended for all dogs, especially those with conditions such as gastrointestinal disturbance, renal and/or liver disease, other comorbidities, increasing age, or undergoing other treatments.

Body weight: ^*^9 out of 9

Refer to the previous stage.

Nutrition/Diet: ^*^9 out of 9

Emphasize the importance of a nutritionally balanced diet.

° Recommend a functional food [e.g., a joint diet containing appropriate quantities of omega-3 essential fatty acids (EFA)].
° If preferred, give omega-3 fatty acid supplements in addition to a normal nutritionally balanced diet.

▪ An Eicosapentaenoic Acid/Docosahexaenoic Acid (EPA/DHA) combined dosage of 75 to 100 mg/kg/day is quoted as a starting or minimum dosage for dogs with OA (30, 31).
▪ The National Research Council (NRC) recommended dose (in mg) for combined EPA/DHA of 310^*^BW^0.75^ for dogs with OA enables a gradual increase in dose if required, but quantities should not exceed the safe upper limit of 370^*^ BW^0.75^ (29, 32). BW is measured in kg.
▪ Any additional benefits of a functional food must also be considered.

° Prioritize omega-3 EFA administration above other nutraceuticals.

Modified Exercise: ^*^9 out of 9

If necessary, adapt breed/life stage/lifestyle-appropriate exercise programs according to clinical OA management requirements.

Involve/refer to a physical therapist/rehabilitation specialist if possible.

Rehabilitation/Physical Therapy: ^*^9 out of 9

Specific rehabilitation/physical therapy protocols are not provided in these recommendations to give maximum flexibility and enable the care plan to be tailored to individual patient needs. Approaches include manual therapies, machinery-applied techniques, and therapeutic exercises. Although the individual treatment modalities may be supported by different levels of evidence, the COAST Development Group recommends that all options should be considered across all clinical stages of OA and that the care plan should be developed by a certified physical therapist/rehabilitation specialist whenever possible.

° Both supervised in-clinic and at-home exercises are highly recommended for strengthening, toning, and improving mobility.
° A certified professional (e.g., acupuncture) can support individually tailored protocols.
° If pet caregiver circumstances are limited (e.g., pet caregiver ability, travel limitations, and financial considerations), work with the physical therapist to determine a solution, rather than eliminating physical therapy from the care plan is recommended. For example, it is often possible to modify the program and/or construct low-cost exercise strategies from items already available at home.

Environmental modifications: ^*^9 out of 9

Consider recommending environmental modifications if problem areas are identified (e.g., hard, slippery floor surfaces).

The extent of changes may depend on the owner's circumstances and their ability to modify the environment appropriately.

- Pharmaceuticals or Biologics

Two different major classes of therapeutics (NSAIDs and an anti-NGF mAb) are approved in many countries for the control of pain in dogs with clinical signs of OA. Both classes of therapeutics have been evaluated for efficacy in similar populations of dogs, are supported by comparable datasets, and currently have similar approvals for use, resulting in the COAST Development Group unanimously recommending the use of either medicine for dogs with clinical signs of OA (Stage 2, 3 or 4). All members of the COAST group agreed on either NSAIDs or anti-NGF as first-line therapy for COAST Stage 3 or 4; 3 of the 9 COAST group members considered an NSAID as first-line therapy for COAST stage 2 dogs, with 6 out of 9 members considering either NSAIDs or anti-NGF as appropriate first-line options for COAST Stage 2. However, there is still much to be understood about how these products will best complement each other within the OA management toolbox, including whether an NSAID and the anti-NGF mAb can be used safely together for the long-term management of dogs with OA pain. There are also differences in mechanisms of action and other factors that may influence product choice, and the COAST Development group encourages consideration of the available information before treatment selection (Appendix 2). Differences in recommendations for product use can occur with geographic expansion and are best reflected in local or regional canine OA treatment guideline adaptations. For more complex clinical scenarios, more defined use of each therapeutic class according to OA stage or OA phenotype might be possible on an internationally relevant basis. Until then, the value of having the choice of different therapeutic options to control pain in dogs with clinical signs of OA should not be underestimated.

Oral products

□ Non-Steroidal Anti-Inflammatory Drugs (NSAID): ^*^9 out of 9

Multiple NSAID products are approved for use in dogs with OA and dosage recommendations correspondingly vary. Most NSAIDs are administered at home, daily, by the oral route, although long duration of action (e.g., 7 days and 1 month) options are available. Many NSAID product labels include “dosage titration,” “intermittent use,” “use at the lowest effective dose,” or similar verbiage. The COAST Development Group supports NSAID dosage reduction or cessation of treatment when appropriate but emphasizes that, if the product is well-tolerated, the dose and the corresponding duration of use should be dependent on the response to treatment and the maintenance of the required level of functional improvement. The main objectives of therapy are to control pain and inflammation and to gradually increase strength, tone, and mobility, so dogs must receive an adequate initial phase of NSAID treatment. The strongest evidence for substantiated efficacy is associated with the use of the recommended therapeutic dose, and ongoing clinical improvement is frequently reported in dogs with OA following several weeks of treatment.

° Consider prescribing a piprant-class, coxib-class, or other cyclooxygenase (COX)-inhibiting NSAIDs for first-line management of pain and inflammation, unless contraindicated.
° Use of the recommended therapeutic dose for a duration of time sufficient to optimize functional improvement is recommended.
° In the experience of the COAST Development group, a minimum of 4 weeks duration of use at the recommended dose is often required for COASTeR Stage 2 dogs.
Evaluate the benefit:risk ratio on an individual case basis and adjust if required.
° A follow-up evaluation (in person or remotely) is advised 7–14 days after first starting treatment to check product tolerability and for early evidence of efficacy (improvement or no deterioration).
° Patient evaluation is required after 1-month of therapy to assess if there is a clinically relevant change and to determine if the use of the NSAID should be extended, if other treatment approaches should be added, or if the NSAID can be stopped or the dosage adjusted due to optimal functional improvement.
° Dogs may respond differently to different NSAIDs. Treatment should be discontinued if no clinical improvement is apparent and other treatment options, including other classes of NSAID, should be explored (see more complex scenarios).
° Regular re-evaluations (e.g., monthly) to monitor response to treatment should be continued for as long as medical management is required. If needed, more frequent re-evaluations (in-person or remotely) can be used to manage dosage regimens. If medical management is stopped, ongoing patient re-visits are important for optimal management of OA but can be less frequent (see COASTeR stage 2 disease education).

Injectable products

□ Anti-NGF Monoclonal Antibody (anti-NGF mAb): ^*^9 out of 9

In countries where the only currently approved canine monoclonal antibody targeting the Nerve Growth Factor (NGF) (bedinvetmab) is available, the administration is recommended at a dose of 0.5–1.0 mg/kg body weight, once every 4 weeks, by the subcutaneous route. The relatively recent introduction of the product limits COAST Group-specific guidance for COASTeR Stage 2 dogs but in a clinical study evaluating dogs with different severities of OA, ongoing clinical improvement was associated with several weeks of treatment. Maximum treatment success was documented ~2 months after the first dose, with a plateau in efficacy thereafter (33). The COAST Development Group therefore currently recommends that if the anti-NGF mAb is well-tolerated, the duration of use should be dependent on the response to treatment and maintenance of the required level of functional improvement.

° As an alternative first-line option for the treatment of canine OA pain, consider administering the anti-NGF monoclonal antibody unless contraindicated.
Note: 3 of the 9 COAST group members consider NSAIDs as first-line therapy for COAST stage 2 dogs, with 6 out of 9 members considering either medication as a potential first-line therapy for COAST stage 2.
° Use of the recommended therapeutic dose for a duration of time sufficient to optimize functional improvement is recommended.

▪ In the experience of the COAST Development group, COASTeR Stage 2 dogs usually require a minimum of 4 weeks of pain control.
▪ Evaluate the benefit:risk ratio on an individual case basis and adjust if required.

° Due to the per-label monthly dosing interval, in-clinic evaluation of the patient just before the administration of a subsequent dose is likely to be most convenient for pet caregivers.

▪ More frequent follow-ups, possibly by remote means of contact, can be used to monitor tolerability and efficacy and discuss pet owner observations.
▪ Evaluate 1 month after the initial dose to assess if there is a clinically relevant change and to determine if treatment use should be extended and if other approaches should be added.

° If there is limited or no response after 1 month of the initial dose of the monoclonal antibody, administration of a second dose is recommended for such dogs, but as with any therapeutic, the product should be discontinued if the patient does not show a positive response after an appropriate time.
° Regular re-evaluations (e.g., monthly) to monitor response to treatment should be continued for as long as medical management is required. If needed, more frequent re-evaluations (in-person or remotely) can be used to address patient requirements. If medical management is stopped, ongoing patient re-visits are important for optimal management of OA but can be less frequent (see COASTeR stage 2 disease education).

*Majority recommendations*: ^**^

- Surgical (vote restricted to orthopedic surgeons only): ^**^ 4 out of 6
- Surgery should be considered to diminish and relieve pain and improve the quality of life of patients that cannot be managed, or managed well-enough, with medical management plus non-drug/non-surgical options.

° Surgical options for dogs with joint disease may be varied and a detailed discussion of these options is beyond the scope of this study. Careful counseling of pet caregivers is recommended and referral to a trained orthopedic surgeon or boarded specialist is recommended where possible.
° Although good to excellent functional outcomes are usually reported for canine patients undergoing accepted surgical procedures, pet caregivers should be aware that patient quality of life can still be compromised post-surgery.
° Surgery may be directed at initiating causes of secondary OA (e.g., arthroscopic removal of osteochrondritis lesions of the shoulder or tarsus), or it may be to treat established OA associated with intractable pain. In some instances (e.g., elbow dysplasia and hip dysplasia), it is recommended that non-surgical/medical management protocols should be explored before considering surgical options, but in other situations (e.g., cranial cruciate ligament rupture, patellar luxation), the indications for surgical options are often clearer.
° Surgery for treating chronic OA associated with intractable pain may include joint replacement, excision arthroplasty, or arthrodesis. The reported outcomes for these techniques vary with the joint affected. At the current time, evidence supports total hip replacement as a good option for suitable subjects (34) but careful counseling of pet caregivers is essential.
  *Minority recommendations*: ^***^

- Non-Drug/Non-Surgical

Nutrition/diet:

□ Supplements

Consider the introduction of an additional supplement either as a single ingredient or as a combination product:

- Chondroitin sulfate: ^***^ 4 out of 9
Glucosamine: ^***^ 4 out of 9
Avocado-soybean unsaponifiables (ASU): ^***^ 4 out of 9
- Undenatured Collagen Type II (UCII): ^***^ 3 out of 9
- Green-lipped mussel: ^***^ 3 out of 9

Factors affecting group decision:

Those not supporting this recommendation felt that clinical studies did not provide enough evidence for the use of the supplement, and/or additional nutritional supplements were not required if the dog's diet was nutritionally balanced, especially if the patient was receiving the other recommendations unanimously supported for COASTeR stage 2. Other concerns included variability in efficacy, additional cost, and variability in the quality of products available.

- Pharmaceuticals or Biologics

Injectable Products:

□ Other:

Consider additional products administered by a veterinarian. The route is product dependent. Referral to centers with extensive experience in intra-articular administrations and familiarity with less well-known treatment modalities is advised:

- Pentosan polysulfate (PPS) (I.M.): ^***^ 3 out of 9
- Polysulfated glycosaminoglycan (PSGAG) (I.M.): ^***^ 3 out of 9
- Low molecular weight hyaluronic acid (HA) (I.A.): ^***^ 1 out of 9.

Factors affecting group decision:

Differences in geographical availability/familiarity with the use of the products, additional costs, and lack of solid scientific evidence and procedural considerations were the main factors limiting the use of a minority recommendation.

**COASTeR Stage 3**

Clinical signs of OA. Moderate osteoarthritis.

*Unanimous recommendations*: ^*^

FOUNDATIONAL ELEMENTS: ^*^9 out of 9

Initiate “all-stage” foundational elements, if not already actioned.

BUILD ELEMENTS

Expand on previous OA stage discussions (COASTeR stages 0–2) by introducing COASTeR Stage 3 applicable topics listed below.

- Non-Drug/Non-Surgical

Disease Education: ^*^9 out of 9

Understanding the fundamentals of the disease and disease progression is relevant for all pet caregivers, but other education requirements will differ if the patient is newly diagnosed or was previously managed and has progressed to this stage of OA.

° Understanding the benefits of multimodal management is important for caregivers of newly diagnosed COASTeR Stage 3 dogs, whereas recognizing the need to adjust the therapeutic approach and potentially alter expectations, may be more important for caregivers of dogs with an established OA management plan.
° COAST staging is required for all newly presented patients or patients that have not been staged previously, to define if any additional investigations are needed to exclude other pathologies (e.g., arthroscopy, CT, MRI) and to confirm OA diagnosis.
° Blood work is recommended for all dogs, especially if there are risk factors for treatment such as gastrointestinal disturbance, renal and/or liver disease, other comorbidities, increasing age, or if the dog is receiving other medications.
° Include a COASTeR Stage 3 treatment-appropriate schedule of visits (e.g., four to six times a year) to increase support relative to the earlier stages of the disease. Due to the need to evaluate response to any changes in treatment, re-visits are likely to be most frequent earlier in the adoption of a COASTeR Stage 3 care plan, although ongoing patient monitoring follow-ups and appointments to drive pet caregiver engagement are also required.

▪ Patient response to new treatment protocols can be variable so highlight the need for flexibility in the visit schedule,
▪ Vet technician/nurse-led and other multi-disciplinary team appointments are strongly encouraged.
▪ The use of engaging educational tools and resources is essential for helping to visualize and simplify more complex management scenarios.

Body weight: ^*^9 out of 9

Refer to the previous stage.

Nutrition/Diet: ^*^9 out of 9

° A joint diet/functional food is recommended for its omega-3 EFA content and other potential benefits (e.g., cartilage health and calorie control).
° If preferred, give an omega-3 fatty acid supplement in addition to a normal nutritionally balanced diet, but ensure a minimum EPA/DHA combined dosage of 100 mg/kg/day. A dosage increase is likely to be required for dogs at this stage of OA, with incremental dose changes (in mg) to NRC canine OA levels of 310^*^BW^0.75^ recommended. The maximal safe limit of 370^*^BW^0.75^ of combined EPA/DHA should not be exceeded. BW is measured in kg.

Any additional benefits of a functional food must be considered.

Modified Exercise: ^*^9 out of 9

Make stage-appropriate adjustments to the exercise plan according to the capabilities of the patient, ideally in partnership with a physical/rehabilitation specialist.

Rehabilitation/Physical Therapy: ^*^9 out of 9

The benefits of referral to a physical therapist/rehabilitation specialist, plus a tailored therapeutic exercise program, should be emphasized. Several members of the COAST Development Group highlighted the potential benefits of machinery-applied techniques for dogs at this stage of the disease.

Environmental modifications: ^*^9 out of 9

Areas of particular focus include comfort (beds and rest areas), non-slip flooring, and facilitating access to high or low areas where required (ramps or steps).

- Pharmaceuticals or Biologics

Refer to the introductory paragraph for COASTeR Stage 2 Pharmaceuticals or Biologics. The COAST Development Group has also provided details of several factors that they feel are important to consider prior to product selection (Appendix 2).

Oral products

□ Non-Steroidal Anti-Inflammatory Drugs (NSAID): ^*^9 out of 9

As for COASTeR stage 2 with the following COASTeR Stage 3 specific details.

° Consider prescribing a piprant-class, coxib-class, or other COX-inhibiting NSAID for the management of pain and inflammation, unless individual patient considerations contraindicate use.
° In the experience of the COAST Development Group, a minimum of 8 weeks duration of use at the recommended therapeutic dose is a realistic initial NSAID requirement for COASTeR Stage 3 dogs, due to the severity of pain and the need to gain better control of the underlying inflammatory and sensitization (hyperalgesia and wind-up) processes, which in turn enables the development of strength and muscle tone.

▪ The total duration of NSAID use should be dependent on the time taken to optimize functional improvement and is subject to patient monitoring and product tolerability.
▪ Key efficacy re-evaluation time points are 1 and 2 months after starting treatment.
▪ More frequent contact (remotely and/or in person), e.g., 7–14 days after first starting treatment, is advised for more comprehensive monitoring purposes.

° If the clinical signs of OA are being managed (to both the clinician's and pet caregiver's satisfaction), and the dog is tolerating the product well, no changes should be made to the protocol before the first key efficacy evaluation time-point (1 month after starting the NSAID). Thereafter, once the benefit of an NSAID has been demonstrated and when an additional functional optimization is needed, additional adjunct analgesia can be considered.
° Use of the NSAID at the recommended therapeutic dose (±adjunct analgesia) should be continued until the required level of functional improvement is achieved.
° Once a satisfactory level of functional improvement has been obtained, protocol adjustment, including NSAID dosage titration, can be considered.

▪ Adjunct analgesia should be ceased before NSAID dosage adjustment downward.
▪ There is no standardized recommendation for titrating down the dose/frequency dose administration for NSAIDs, but the COAST Development Group suggests extending the administration interval in preference to lowering the dose.
▪ To minimize the risk of suboptimal pain relief, regular re-evaluation of the patient is required, utilizing validated efficacy evaluation tools whenever possible and considering both veterinary and pet caregiver observations.
▪ In some cases, cessation of NSAID treatment may be possible.

° Ongoing requirements for anti-inflammatory pain relief should be assessed on an individual basis, guided by evaluations of therapeutic response and NSAID tolerability.

Injectable products

□ Anti-NGF Monoclonal Antibody: ^*^9 out of 9

As for COASTeR Stage 2 with the following COASTeR Stage 3 specific details.

° Consider administration of the canine monoclonal antibody targeting NGF, unless contraindicated.
° In the experience of the COAST Development Group, the control of pain, especially the underlying sensitization processes, thereby enabling the development of strength and muscle tone in COASTeR Stage 3 dogs, is likely to require a minimum of 8 weeks of pain relief. However, this is based on clinical anecdotal experience, and individual variability may occur (i.e., clinical improvements might be observed before or after the 8 weeks).

▪ The total duration of anti-NGF mAb use should therefore be dependent on the time taken to optimize functional improvement and is subject to patient monitoring and product tolerability.
▪ Key efficacy evaluation points are 1 and 2 months after starting treatment.
▪ Coordinating follow-up evaluations just before the administration of the next dose is likely to be most convenient for pet caregivers.
▪ More frequent follow-ups (remotely and/or in person), e.g., 7–14 days after first starting treatment, may be utilized for more comprehensive patient monitoring.

° Subject to the clinical signs of OA being managed (to both the clinician's and pet caregiver's satisfaction), and the dog tolerating the product well, no changes should be made to the protocol before the first key efficacy evaluation time-point (1 month after starting the mAb). Thereafter, once the benefit of the mAb has been demonstrated but when additional functional optimization is needed, additional adjunct analgesia can be considered.

▪ Due to the relatively recent introduction of this product, the COAST group's experience of use with an adjunct analgesic is limited. Benefit:risk evaluations are advised when any products are used concurrently.

° Use of the mAb at the recommended therapeutic dose (± adjunct analgesia) should be continued until the required level of functional improvement is achieved. Once obtained, protocol adjustment may be considered.

▪ Adjunct analgesic(s) should be ceased before any adjustment of mAb administration.
▪ There is currently no data or recommendations to guide decisions around mAb dosage adjustment.
▪ To minimize the risk of suboptimal pain relief, regular re-evaluation of the patient is required, utilizing validated efficacy evaluation tools whenever possible and considering both veterinary and pet caregiver observations.
▪ In some cases, cessation of mAb treatment may be possible.

° Ongoing requirements for pain relief should be assessed on an individual basis, guided by evaluations of therapeutic response and product tolerability.

- Surgical (vote restricted to orthopedic surgeons only): ^*^6 out of 6
- Refer to COASTeR Stage 2. The increased demands of more complex medical management protocols in COASTeR Stage 3 may influence decision-making more strongly.

*Majority recommendations*: ^**^

- Pharmaceuticals or Biologics

Oral Products

□ Adjunct Analgesics
- Amantadine^†♢^: ^**^8 out of 9
- Acetaminophen: ^**^7 out of 9
- Gabapentin^†^: ^**^6 out of 9

For patients improving on a unanimously recommended pain control option but still requiring greater pain relief, the majority of the COAST development group recommends considering the addition of an adjunct analgesic into the pain management protocol. This is particularly important for dogs with, or suspected of having, sensitization and/or chronic neuropathic pain.

° The emphasis is on the addition of analgesia and not the replacement of the NSAID or the mAb by an adjunct analgesic.
° The introduction of an adjunct analgesic can be considered during the first 2 months of NSAID or mAb treatment if required.

▪ To confirm the clinical benefit of the primary analgesic before the addition of an adjunct analgesic, the adjunct should be introduced following the first key efficacy re-evaluation time point (e.g., 1 month after starting the first treatment).
▪ The adjunct analgesic may be continued if required but should be stopped before dosage adjustment/titration of the primary analgesic.

° If necessary, more than one adjunct analgesic can be incorporated into the protocol in a stepwise fashion, but the clinical benefit of each should be demonstrated before the addition of the next.

Factors affecting the group decision:

Dosing variability (different dosages evaluated in published studies); likelihood of delayed onset of action of some products; challenges associated with multiple drug administration (cost, compliance, demonstration of efficacy, and potential adverse effects); not all members of the group had extensive experience with all products (geographical differences).

- ◇ Important note: In May 2022, the European Medicines Agency released a document for consultation proposing the reservation for human use of some antivirals, including amantadine. The proposal was published due to the limited treatment options for specific viral diseases in human medicine and an evaluation of the potential risk of selection and dissemination of resistant viruses to humans, from the use of antiviral substances in animals.
- Due to the good quality evidence of the efficacy of amantadine in animals for analgesia, particularly its use in animals with central sensitization and hyperalgesia, as well as the challenges of finding alternative medications for adjuvant chronic pain therapy, the COAST development group has included amantadine in its recommendations. The COAST group acknowledges that if a decision is made to reserve the use of amantadine for human medicine, it will not be possible to continue to recommend it as an adjunct analgesic for dogs with OA in those geographies.

Injectable products

□ Other:

Consider products administered by the intra-articular (I.A.) route if further functional improvement is required following the administration of unanimously recommended treatment modalities and/or if adverse effects, comorbidities, or other medical considerations limit other treatment options.

- Stem cells (I.A.): ^**^5 out of 9
° Working with/referral to centers with experience in this approach, utilizing licensed laboratories, and adhering to strict quality standards are recommended to support product consistency, safety, and efficacy.
° Regulatory policies applicable to stem cell products are present in some countries and should be adhered to.

Factors affecting the group decision:

Not all of the COAST group had extensive personal experience with this treatment option. The evidence of efficacy is limited/equivocal, in part due to this approach still being in its relative infancy and there being substantial diversity of stem cell products including in cell origin, processing methods, and application methods. Each stem cell product should be evaluated in isolation. Cost and procedural considerations also raise some concerns.

*Minority recommendations*: ^***^

- Non-Drug/Non-Surgical options

Nutrition/Dietary:

□ Supplements:

Consider the introduction of an additional supplement either as a single ingredient or as a combination product, although this is unlikely to be a requirement if the patient is already receiving a joint diet/functional food containing the same or similar ingredients:

- Chondroitin sulfate: ^***^3 out of 9
- Glucosamine: ^***^3 out of 9
- Avocado-soybean unsaponifiables (ASU): ^***^3 out of 9
- Undenatured Collagen Type II (UCII): ^***^3 out of 9
- Green-lipped mussel: ^***^3 out of 9
- Cannabidiol (CBD) supplement: ^***^3 out of 9.

Factors affecting the group decision:

Limited evidence base/equivocal efficacy, familiarity, associated additional costs, and variability in product quality between suppliers limited these supplements to a minority recommendation.

- Pharmaceuticals or Biologics

Oral products

□ Adjunct Analgesics
- Tramadol: ^***^1 out of 9

Other adjunct analgesics are more strongly recommended for use in dogs and should be used in preference to tramadol whenever possible (refer to majority recommendations). Potentially consider the use of tramadol as an additional adjunct when more aggressive use of analgesics may be required, such as for recurrent or relapsed cases.

Evidence of the efficacy of oral tramadol alone in dogs with OA, as demonstrated by a reduction in signs of pain or an improvement in orthopedic function, is lacking (35). Use as an adjunct analgesic is a consideration because of possible relevance in the control of the affective component of pain (i.e., norepinephrine and serotonin reuptake inhibition) and potential co-administration synergism with NSAIDs, gabapentin, amantadine, or opioids, although evidence of a beneficial synergistic effect of tramadol in dogs with OA is limited (36).

Factors affecting the group decision:

Questionable efficacy due to the limited production of the active metabolite of tramadol in dogs based on current literature, challenges associated with the administration of multiple drugs, and lack of extensive in-use experience of this treatment option in some geographies.

Injectable products

□ Other:

Consider other products administered by a veterinarian. The route is product-dependent (see below). Referral to centers with extensive experience in intra-articular administrations and familiarity with less well-known treatment modalities is advised.

- Low molecular weight hyaluronic acid (HA) (I.A.): ^***^4 out of 9
- Pentosan polysulfate (PPS) (I.M.): ^***^ 3 out of 9
- Polysulfated glycosaminoglycan (PSGAG) (I.M.): ^***^ 3 out of 9
- Platelet Rich Plasma (I.A.): ^***^2 out of 9
Sourced from licensed laboratories and adhering to strict quality standards is recommended to support product consistency, safety, and efficacy.
- Corticosteroids (I.A.): ^***^2 out of 9
Consider cases that are difficult to manage or when other treatment options are limited or have failed:

Factors affecting group decision:

Differences in geographical availability/familiarity with the use of the products, additional costs, and procedural considerations were the main factors limiting the use of a minority recommendation.

The minority consensus was that potential disease-modifying effects of PPS/PSGAG which were more relevant in the earlier stages of the disease.

**COASTeR Stage 4**

Clinical signs of OA. Severe osteoarthritis.

*Unanimous recommendations*: ^*^

FOUNDATIONAL ELEMENTS: ^*^9 out of 9

Initiate “all-stage” foundational elements, if not already actioned.

BUILD ELEMENTS

Expand on previous OA stage discussions (COASTeR stages 0–3) by introducing COASTeR Stage 4 applicable topics listed below.

- Non-Drug/Non-Surgical

Disease Education: ^*^9 out of 9

Education requirements will differ if the patient is newly diagnosed or was previously managed and has progressed to this stage of OA.

Dogs at this stage of OA can differ notably from each other in terms of clinical signs, ranging from being seriously incapacitated to being completely unable to move. All are severely affected by OA.

° Ensure that the pet caregiver understands the severity of their dog's condition and the need for a rapid introduction of both pharmaceutical and non-pharmaceutical treatment modalities.
° A COAST staging is required for all newly diagnosed patients or patients that have not been staged previously. Radiography must be included to confirm OA diagnosis, exclude any other pathologies, and define if any additional investigations are needed.
° Blood work is recommended for all dogs. Many dogs at this stage of the disease are elderly and comorbidities are also likely. However, the need to control severe OA pain because of the quality-of-life implications will have a greater influence on benefit:risk evaluations. A thorough assessment of each patient is compulsory to determine how aggressive pain management protocols can be while still considering patient safety.
° For newly diagnosed cases, explain the need for multi-modal therapy and the reasons for a more rapid introduction of pharmacological adjuncts, when the patient's overall health status allows.
° Remaining management options may be limited if the patient has already received an extensive care plan during earlier stages of the disease. Help pet caregivers understand what additional medical management approaches are available and consider surgical options if appropriate.
° Frequent follow-ups will be required for COASTeR Stage 4 dogs, because of the more complex medical situation and for patient welfare monitoring.

▪ Weekly updates during the initial period of the care plan should be an expectation. Telehealth and videos of the dog in the home environment can help to reduce the burden associated with the transport of the patient to the clinic.
▪ Pet caregivers must understand the need for a regular hands-on evaluation of the dog (e.g., every month) until sufficient functional improvement is confirmed.
▪ Vet technician/nurse-led and other multi-disciplinary team appointments are strongly encouraged.
▪ Engaging educational tools and resources is crucial for helping to visualize and simplify complex management scenarios at stressful times. They are also useful memory aids, enabling pet caregivers to reflect and reconsider important information.
▪ Incorporate at-home visits if required/possible.

° A COASTeR Stage 4 care plan should include end-of-life preparation (support for the patient and pet caregiver)

Body weight: ^*^9 out of 9

Excess body weight or obesity is often a factor in dogs with severe OA. Its contribution to joint pain and limited mobility makes weight management an important but challenging part of COASTeR stage 4 OA protocols. Due to disease severity, initial discussions and care plans must focus on the urgent requirements of Stage 4 patients, such as improving patient comfort as rapidly as possible. Body weight management can be carefully introduced as part of this initial plan if care is taken not to overwhelm the pet caregiver with information and too many instructions. The emphasis on weight management can be gradually increased once OA pain is better controlled.

Nutrition/Diet: ^*^9 out of 9

Like weight management discussions, nutrition and dietary requirements should be incorporated into COASTeR stage 4 OA management protocols but should be prioritized after urgent care considerations have been addressed. Dogs progressing from medical management of COASTeR stage 3 are probably receiving functional food and/or nutritional supplements.

Exercise: ^*^9 out of 9

There is a range in physical ability at this stage of OA, but in general, pet caregivers are likely to be concerned about the significant impact of the disease on their dog's ability to exercise. Individual patient evaluation is particularly important to ascertain the current level of mobility and determine an appropriate exercise goal.

Rehabilitation/Physical therapy: ^*^9 out of 9

The potential benefits of rehabilitation/physical therapy as part of a multi-modal OA management approach must be emphasized. Physical therapy programs offer multiple different treatment options, making them suitable for complex medical cases. Pet caregivers should be informed about mobility assistance devices so that they can make an educated choice (refer to the Palliative care section).

Environmental modifications: ^*^9 out of 9

Environmental modifications are crucial for COASTeR Stage 4 dogs. Areas of particular focus include

° Comfort (beds and rest areas).
° Reducing the distance and effort needed to travel to important areas, such as for eating, drinking, and toileting.
° Facilitating access to any important areas with ramps or other support elements.
° Increasing traction offered by walking surfaces with the use of non-slip flooring.

- Pharmaceuticals or Biologics

As for COASTeR Stage 3 but with the addition of COASTeR Stage 4 specific details. A more aggressive use of analgesics with a more rapid introduction of pharmacological adjuncts is recommended for COASTeR Stage 4 dogs. Although it is not known if an NSAID and the anti-NGF mAb can be used safely together for the long-term management of dogs with OA, the benefits of the differences in mode of action may necessitate a benefit:risk analysis for concurrent use in dogs where pain management is particularly challenging (refer to Appendix 2 for considerations prior to product use).

Oral products

□ Non-Steroidal Anti-Inflammatory Drugs (NSAID): ^*^9 out of 9.

° Consider prescribing a piprant-class, coxib-class, or other COX-inhibiting NSAID for the management of pain and inflammation, unless individual patient considerations contraindicate use.
° In the opinion of the COAST Development Group, a minimum of 12 weeks duration of NSAID use at the recommended therapeutic dose is an expectation for COASTeR Stage 4 dogs. It is required to gain functional improvement through better control of underlying pain sensitization processes and improved strength.

▪ Due to disease severity, the increased probability of comorbidities, and other medical considerations, COASTeR Stage 4 dogs, will require frequent monitoring (see disease education).
▪ Key efficacy evaluation time points (preferably hands-on) are 1, 2, and 3 months after treatment initiation.

° Subject to tolerability assessments, continue NSAID use at the recommended therapeutic dose, for as long as is required to obtain and maintain the required level of functional improvement; several NSAIDs are approved for long-term use for canine musculoskeletal disease (OA).

1. ▪ NSAID requirements of dogs with advanced OA are often lifelong.
2. ▪ NSAID dosage reduction may be possible with care and frequent monitoring but should not be an expectation.
3. ▪ Complete cessation of NSAID use is unlikely at this stage of OA.

° If clinical improvements are seen but additional pain relief is required:

▪ Consider a more rapid/proactive introduction of adjunct analgesics, e.g., at the 1-month efficacy re-evaluation.
▪ Do not stop the NSAID, unless there is a medical reason for doing so.
▪ Adjunct dose increases and/or the relatively rapid introduction of another adjunct analgesic may be required (it usually takes at least 3 to 4 weeks to confirm a benefit of any prior introduced adjunct analgesics).
▪ Benefit: risk evaluations are advised when any product is used concurrently.

° If the NSAID is well-tolerated but there is no clinical improvement or deterioration is evident, stop the NSAID.

▪ At this stage of the disease and in this situation, the benefit of switching to another NSAID is limited, unless there is an obvious medical reason for doing so.
▪ Instead, consider other analgesic options and management approaches.

Injectable products

□ Anti-NGF Monoclonal Antibody: ^*^9 out of 9

° Consider administering a canine monoclonal antibody targeting NGF, unless contraindicated.
° In the experience of the COAST Development Group, better control of pain sensitization and an improvement in the strength of COASTeR Stage 4 dogs is likely to require a minimum of 12 weeks (3 months) of pain relief. However, individual response and duration of use may vary, and optimization of functional improvement should determine the duration of anti-NGF mAb use.

▪ COASTeR Stage 4 dogs require frequent monitoring due to disease severity, the increased probability of comorbidities, and other medical considerations (see disease education).
▪ Key efficacy evaluation points (preferably hands-on) are 1, 2, and 3 months after starting treatment, although more frequent general follow-ups may be of benefit.

° Subject to tolerability assessments, continue anti-NGF mAb use at the recommended therapeutic dose, for as long as is required to obtain and maintain the required level of functional improvement.

▪ Pain control requirements of dogs with advanced OA are often lifelong.
▪ Reduction in dosing frequency may be possible with care and frequent monitoring, but it should not be an expectation.
▪ Complete cessation of mAb use is unlikely at this stage of OA.

° Due to the relatively recent introduction of this product, the COAST group's experience using the mAb with one or more adjunct analgesics is limited. If the mAb is well-tolerated, and clinical improvements are seen but additional pain relief is required, the patient may benefit from the more rapid administration of one or more adjunct analgesics.

▪ A benefit:risk evaluation is advised when any products are used concurrently and is important when the experience of concomitant administration is low.
▪ Consider a more rapid introduction of adjunct analgesics (e.g., at the 1-month efficacy re-evaluation).
▪ Do not stop the mAb unless there is a medical reason for doing so.
▪ Adjunct dose increases and/or the relatively rapid introduction of another adjunct analgesic may be required (it usually takes at least 3 to 4 weeks to confirm a benefit of any prior introduced adjunct analgesic).

° If the mAb is well-tolerated but there is no clinical improvement, or deterioration is evident, do not repeat the administration.

▪ Some dogs do not respond or have a limited response to the first injection.
▪ At this stage of OA, alleviating the pain is a priority.
▪ Consider other analgesic options and management approaches.

□ Other:
- Stem cells (I.A.): ^*^9 out of 9

° Refer to COASTeR Stage 3.
° Commitment to high-quality standards is mandatory.
° Referral to centers with extensive experience in this approach is recommended.
° Adhere to any geographically applicable regulatory policies.

Factors affecting group decision:
As for COASTeR Stage 3, the main factors were a lack of extensive in-use experience with this treatment option in some geographies, equivocal evidence of efficacy, and concerns about costs and quality standards. The group felt that for dogs with severe, late-stage osteoarthritis, the potential benefit was greater than the risk.

- Surgical (vote restricted to orthopedic surgeons only): ^*^6 out of 6

° Refer to COASTeR Stage 3.
° Poor physical condition and/or other factors such as comorbidities may influence surgical option decision-making.

▪ Extensively explore medical management options including majority and minority recommendations.
▪ Seek specialist advice before surgery. Referral is always an option.

*Majority recommendations*: ^**^

- Non-Drug/Non-Surgical

Nutrition/Dietary

□ Supplement:
- Cannabidiol (CBD) supplements: ^**^6 out of 9

Factors affecting the group decision:
The group had concerns about the limited evidence base/equivocal efficacy, associated additional costs, and variability in product quality between suppliers. However, adjunct pain relief is a significant requirement in dogs with COASTeR stage 4 OA, and preliminary data indicate that CBD has the therapeutic potential to contribute to pain management in dogs with OA (37). The possible role of CBD in enhancing the effect of concurrent analgesic drugs and improving pain support in dogs with chronic maladaptive pain increases the strength of the recommendation for use in dogs with severe OA.

- Pharmaceuticals or Biologics

Oral products

□ Adjunct analgesics.

° Consider the introduction of pharmacological adjuncts in a stepwise manner.
° Re-evaluation is important to demonstrate efficacy and tolerability and ascertain if additional analgesia is required.
° Patients may require multiple adjunct analgesics at this stage of the disease.

- Amantadine^†^: ^**^8 out of 9
Note: European Medicines Agency consultation document proposing the reservation for human use of some antivirals, including amantadine (Refer to COASTeR Stage 3 for details).
- Gabapentin^†^: ^**^8 out of 9
- Acetaminophen: ^**^7 out of 9
- Tramadol: ^**^6 out of 9

Factors affecting the group decision:

Dosing variability (different dosages evaluated in published studies); likelihood of delayed onset of action of some products; challenges associated with multiple drug administration (cost, compliance, demonstration of efficacy, and potential adverse effects); not all members of the group had extensive experience with all products (geographical differences).

Additional concerns about weaker evidence-based efficacy (tramadol; see COASTeR Stage 3) or safety as a long-term adjunct to NSAIDs (acetaminophen) meant that these two options received less support than amantadine and gabapentin.

Injectable products

□ Other:

If only one or two joints are affected (but severely), intra-articular injections should be a key consideration for Stage 4 dogs.

- Corticosteroids (I.A.): ^**^8 out of 9

° The use of NSAIDs with steroids is contra-indicated and the withdrawal of NSAIDs for 1 week, to enable steroid administration, should be evaluated. However, the severity of OA and the need for ongoing analgesia is a significant factor in the benefit:risk evaluation for COASTeR stage 4 dogs.

- Platelet Rich Plasma (I.A.): ^**^7 out of 9

° Sourced from licensed laboratories and adhering to strict quality standards is recommended to support product consistency, safety, and efficacy.

- Hyaluronic acid (HA) (I.A.): ^**^6 out of 9

° Restrict use to low molecular weight HA if this product has not been implemented as part of an earlier COAST stage management protocol.
° Consider the use of both low and high molecular weight HA for dogs where low molecular weight HA has been used previously.

- Pentosan polysulfate (PPS) (I.M.): ^**^ 6 out of 9
- Polysulfated glycosaminoglycan (PSGAG) (I.M.): ^**^ 6 out of 9

Factors affecting the group decision:c
Corticosteroids have an established efficacy profile, are a cost-effective option, and there is familiarity with their intra-articular use in most geographical areas.
Low molecular weight HA has less risk of adverse effects compared to high molecular weight HA. High molecular weight HA can be associated with increased fluid retention in the joint and may be detrimental to cartilage but potential benefits, such as increased synovial fluid viscosity and strong anti-inflammatory effect, may provide functional improvement in dogs needing more aggressive medical support. The potential disease-modifying effects of PPS or PSGAG are likely to be more relevant in the earlier stages of OA, although the possibility of reparative effects in severely damaged joints makes these products of some interest in COASTeR stage 4 dogs.

*Minority recommendations*: ^***^

- Non-Drug/Non-Surgical

Nutrition/diet

□ Supplements:
- Chondroitin sulfate: ^***^3 out of 9
- Glucosamine: ^***^3 out of 9
- Avocado-soybean unsaponifiables (ASU): ^***^3 out of 9
- Undenatured Collagen Type II (UCII): ^***^3 out of 9
- Green-lipped mussel: ^***^3 out of 9

Factors affecting the group decision:
Limited evidence base/equivocal efficacy, variability in quality between suppliers, and additional costs were the main concerns. Patients are receiving multiple drugs at this stage of the disease and dietary supplements were considered of lesser importance vs. other treatment modalities.

### More complex scenarios: NSAID lack of tolerability or efficacy

Applies to clinical COAST stages of OA (COASTeR Stages 2, 3 & 4)

*Unanimous recommendations*: ^*^

- Pharmaceuticals or Biologics

Oral products

□ Non-Steroidal Anti-Inflammatory Drugs (NSAID): ^*^9 out of 9

If a patient has had an inadequate response to an NSAID or developed an adverse event, it is recommended to try another NSAID after a suitable washout period. Adverse effects or lack of efficacy with one NSAID does not necessarily mean intolerance or lack of efficacy with all NSAIDs. Safety is paramount, and patients must be closely monitored.

° Ideally select a class (piprant, coxib, and preferential COX inhibitor) that differs from the previous NSAID used.
° Consider the benefits:risks of each mode of action.
° When switching between NSAIDs, when no side effects have been seen, a washout period of 5–7 days minimizes the chances of adverse drug interactions (38). A washout period of 2 to 3 days may be considered sufficient for contemporary NSAIDs, although the pharmacological profile of each product and the individual and any clinical indicators of ongoing NSAID action should be considered.
° A longer washout period (e.g., 7–10 days, but appropriate for the side effect experienced by the dog) should be allowed in patients with adverse events. It is particularly important to make sure that the previous NSAID has been eliminated from the body and that any side effects have been resolved.
° Respectively, longer washout periods should be considered for extended duration of action products.
° Opinions on how to determine NSAID washout periods differ but calculations based on drug elimination half-life are frequently proposed. Some consider the probability of drug interactions to be minimal after the expiration of three to four half-lives, although more conservative estimates recommend that 5× to 10× the elimination half-life should be allowed (38). Unfortunately, NSAID half-lives are variable and tissue effects are not necessarily linked with plasma half-life. Washout times should therefore also consider physiologic carryover effects or other factors influencing ongoing activity such as prolonged tissue binding.
° Alternative analgesics should be provided during the wash-out period.
° The use of an NSAID with a gastro-protectant is not advocated (39). The need for a gastro-protectant is an indication that the NSAID is not well-tolerated.

If the patient is confirmed NSAID intolerant (adverse effects experienced with separate use of two different NSAIDs), cessation of NSAID use is required and other types of analgesic should be considered instead.

° Refer to other analgesic recommendations in the COAST treatment guidelines, especially the pharmaceuticals and biologics sections.
° It may be necessary to refer to higher COASTeR stages for a more complete list of recommendations.
° Due to its different mechanisms of action, patients may be able to tolerate acetaminophen, even if they are NSAID intolerant.
° Local administration (e.g., intra-articular) of appropriate products may help to improve the comfort of patients particularly prone to side effects with systemically administered drugs (refer to Injectable products sections). Adjuvant analgesics might be given for pain relief if NSAIDs are not tolerated.

### More complex scenarios: severe “acute-on-chronic” or breakthrough pain

Applies to all clinical COAST stages of OA (COASTeR Stages 2, 3 & 4) but this section is particularly relevant for COASTeR stage 4 patients. Consider short-term administration of injectable drugs (e.g., opioid, ketamine, and/or lidocaine infusions) in the hospital setting to manage cases of breakthrough or “acute on chronic” pain that has not been managed effectively with more routine medical management options.

*Unanimous recommendations*: ^*^

- Pharmaceuticals or Biologics

Injectable products

□ Other
- Ketamine (I.V. infusions): ^*^9 out of 9
- Lidocaine (I.V. infusions): ^*^9 out of 9

*Majority recommendations*: ^**^

Pharmaceuticals or Biologics

Injectable products

□ Other:
- Opioids (I.M.): ^**^8 out of 9
- Opioids (I.V. CRI): ^**^8 out of 9

### More complex scenarios: palliative care/end-of-life management

*Unanimous recommendations*: ^*^

- Non-Drug/Non-Surgical

Disease Education: ^*^9 out of 9
This is a particularly stressful time and ensuring pet caregivers are fully informed, supported, and gradually prepared for their pet's end-of-life is the optimal goal.

° Veterinary technician/nurse-led and other multi-disciplinary team appointments are strongly encouraged to enable more in-depth discussions and to address questions or concerns.
° Accommodate pet caregiver wishes whenever possible.

Mobility assistance devices: ^*^9 out of 9
Mobility assistance devices are a consideration for dogs with severe OA (COASTeR Stage 4) and may be of value for other dogs depending on their circumstances.

° Slings/carry bags or ambulation carts provide extra support.
° Orthotic braces may help stabilize joints in particular circumstances.

Although they may not be appropriate for all patients or circumstances, wheeled mobility devices for dogs (e.g., wheelchairs, stroller/ pram, or trailers) may be considered if the pain is controlled at rest but not when mobile.

- Pharmaceuticals or Biologics

Oral products
- Corticosteroids: ^*^9 out of 9

° Recommended for end-stage management only.
° Consider ONLY if all other options have failed to manage pain sufficiently.
° Use “instead of” rather than in addition to other analgesics and consider other salvage options if applicable (refer to surgical options).
° Make pet caregivers aware of common adverse effects that may impact home care.

### Not currently recommended for the management of canine OA:

- Pharmaceuticals or Biologics

From the evaluated list of products and approaches, the COAST Development Group does not currently support the use of the following items for the management of dogs with OA, at any stage of the disease.
Oral products

□ Adjunct Analgesics

- Opioids (P.O.)

Factors affecting group decision:
At-home safety (accidental ingestion or misuse), stability of dispensed products, high first-pass effects in dogs after oral administration, and questionable efficacy of non-approved routes of administration or formulation.

Injectable products

□ Other:
- Botulinum toxin (I.A.)

Factors affecting group decision:
Despite some positive data in humans, there is a lack of/conflicting evidence for efficacy in dogs.

## Discussion

The COAST development group recommendations for the management and treatment of canine OA are the first proposed internationally applicable guidelines based on the COASTeR stage. Optimal management of OA is complex, requiring the consideration and selection of multiple intervention options (both pharmaceutical and non-pharmaceutical). Similarities in OA stage-specific management protocols are likely, but care plans may vary between dogs or may change during the treatment of an individual dog, depending on specific medical needs and response to treatment. The COAST group consensus is intended to support veterinarians with the development of effective OA care plans by providing a practical and evidence- and clinical experience-based reference of stage-specific management options. By utilizing the flexible “base and build” approach, the authors believe that it is possible to develop distinct protocols for a diverse population of pre-clinical or clinically affected dogs. Division of the COAST group recommendations into unanimous, majority, and minority categories encourages the selection of management options to strengthen the evidence, but the inclusion of COAST group votes and an explanation of factors leading to those votes provide additional context for benefit:risk evaluations in more complicated scenarios.

The use of COAST staging in the development of the treatment guidelines encourages the evaluation and management of pre-clinical dogs and dogs with clinical signs of OA. As a result, the COAST group consensus highlights the need for optimization of body weight, body condition, muscle strength and tone, exercise, and nutrition, to help avoid or mitigate OA risk factors or to positively contribute to clinical OA management programs. Another subject of COAST group emphasis is the requirement for an ongoing, consistent approach to pet caregiver education. Providing information in an easy-to-understand but engaging format, and incrementally building the content over time, can help gain and sustain pet caregiver engagement and encourage pet owners to become an integral and effective part of their dog's care team. For dogs with clinical signs of OA, the guidelines reflect the need to consider underlying pathophysiologic processes as a means of optimizing therapy. In particular, the COAST development group underlines the need for effective pain control to support patient comfort and quality of life and to facilitate rehabilitation programs within a multidisciplinary approach. Regular re-evaluation of patients is encouraged to monitor both efficacy and tolerability and to support the administration of proven efficacious dosages for the duration necessary to achieve the desired functional outcome.

To promote easy reference, the COAST treatment recommendations for canine OA have been provided by the COASTeR stage. However, the need to include explanatory details and to re-iterate some information between stages extends the length of this primary reference document. It is envisioned that familiarity with the recommendations will ease and quicken the use of the guidelines, and supportive tools will improve practical utilization. Veterinarians are also encouraged to use the most up-to-date information available. Over time, it is expected that the treatment guidelines will be revised and updated to reflect product innovation and new study data. In addition, local or regional differences in product approval and familiarity with or acceptance of certain treatment modalities or veterinary techniques are likely to drive the development of country- or region-specific treatment recommendations for dogs with OA. These local adaptations are encouraged and will complement the international treatment guidelines by providing more detail about geographically specific approaches and opinions. As mentioned before, these guidelines are consensus-based and supported by literature evidence. However, a detailed and extensive systematic review and/or meta-analysis of results for each type of intervention and/or therapy using specific reporting guidelines (e.g., PRISMA, COSMIN, etc.) was not attempted but could be incorporated into future guidelines. The authors recognize the importance of more in-depth investigations using such comprehensive methodologies which would certainly strengthen evidence and provide valuable information for treatment rationale in future updates of this document. However, there is extremely little “stage-specific” information on any of the available treatment options, limiting the impact of this approach in the context of acknowledging treatment recommendations vary with disease burden and impact.

Unfortunately, the treatment recommendations address only one aspect of the challenges facing veterinarians looking to improve the welfare of dogs with or at risk of OA. Although canine OA is primarily caused by developmental abnormalities of the joints and has been reported to clinically affect approximately one-quarter of younger dogs (8 months to 4 years of age) (40), young dogs are not a common focus group for regular osteoarthritis monitoring and pet caregivers may not be aware of, or may struggle to see, more subtle signs of mobility impairment. It is hoped that the availability of the treatment guidelines will further elevate the use of the COAST Staging tool, helping to increase pet caregiver acceptance of pro-active OA evaluations in younger dogs and empowering veterinarians to drive diagnosis of canine OA earlier in the course of the disease. It is possible that the reduction or elimination of OA risk or progression factors, and the earlier optimization of care plans, could slow disease progression but, as a minimum, it should lead to a greater number of dogs receiving a more sustained approach to OA management that gradually expands over time. This should help to reduce the stressful and unsatisfactory “fire-brigade” approach that is all too often required when dogs are first diagnosed when they already have advanced signs of OA.

Finally, the authors appreciate that canine OA management is not a “one-size fits all” approach. The consensus provides recommendations and guidance on staging using respective foundational and build elements for non-pharmacological and pharmacological approaches. However, some case scenarios and responses to treatment and efficacy may differ among individuals. Therefore, the document should be used for clinical decision-making but always taking into consideration that each patient is unique. Clinical judgment is warranted and should be considered according to patient condition, financial and expertise constraints, treatment familiarity and availability, and comorbidities, among other factors. The guidelines may also be used to indicate gaps of knowledge in the field of canine OA that could instigate further studies and continuing education in the subject. Future COAST validation studies will be also important to corroborate our consensus approach based on staging.

## Conclusion

Canine osteoarthritis is a complex disease and only animal healthcare professionals with personal knowledge of the patient can optimize care plans to meet the needs of the patient and requirements of the pet caregiver. This proposal for the first international guidelines for the treatment of canine osteoarthritis (OA), according to the COASTeR OA stage, is intended to provide a practical reference to evidence-based recommendations and expert opinion while leaving decision-making and the development of protocols appropriate to each dog's specific situation, firmly in the hands of the consulting veterinarian. The authors encourage the use of the COAST staging tool and the COAST canine OA treatment guidelines proposal, and welcome feedback to help guide future updates and the optimization of both (coastdevgroup@gmail.com).

## Author contributions

All authors listed have made a substantial, direct, and intellectual contribution to the work and approved it for publication.
