# Supplementary material for: COAST Development Group's international consensus guidelines for the treatment of canine osteoarthritis
Source: Front Vet Sci. 2023 Aug 3;10:1137888. doi: 10.3389/fvets.2023.1137888 (PMC10436090; doi:10.3389/fvets.2023.1137888)
Supplement: Supplementary file 1 [file Data_Sheet_1.pdf]

Appendix 1: Canine osteoarthritis treatment modalities and management approaches evaluated by the COAST Development Group during development of the treatment guidelines

<table>
<tr><td rowspan="16">Non-drug / non-surgical</td><td>Pet caregiver education</td><td></td></tr>
<tr><td>Body weight optimization</td><td></td></tr>
<tr><td>Appropriate exercise</td><td></td></tr>
<tr><td>Nutrition/dietary</td><td></td></tr>
<tr><td>Environmental modifications</td><td></td></tr>
<tr><td>Mobility Assistance Devices</td><td></td></tr>
<tr><td rowspan="3">Rehabilitation/ Physical therapy/</td><td>Manual Therapy: Examples: Cryotherapy/Thermotherapy, Massage, Myofascial release, Range of motion (passive and assisted), Traction</td></tr>
<tr><td>Movement & exercise: Hydrotherapy, Proprioceptive Exercise, Therapeutic Exercise, Treadmill</td></tr>
<tr><td>Machinery or Instrument applied: (Electro)Acupuncture, Electrical Nerve Stimulation, Extracorporeal Shockwave therapy, Photobiomodulation, Pulsed electromagnetic field therapy, Ultrasound</td></tr>
<tr><td rowspan="7">Oral Supplements</td><td>Omega-3 EFA</td></tr>
<tr><td>Chondroitin sulphate</td></tr>
<tr><td>Glucosamine</td></tr>
<tr><td>ASU</td></tr>
<tr><td>Green lipped mussel</td></tr>
<tr><td>Undenatured Type II collagen (UCII)</td></tr>
<tr><td>Cannabinoid (CBD) supplements</td></tr>
<tr><td rowspan="8">Oral drugs</td><td rowspan="2">NSAIDs</td><td>COX inhibitors</td></tr>
<tr><td>Piprants</td></tr>
<tr><td>Corticosteroids</td><td></td></tr>
<tr><td>Amantadine</td><td rowspan="5">Adjunct analgesics</td></tr>
<tr><td>Gabapentin</td></tr>
<tr><td>Acetominophen</td></tr>
<tr><td>Opioids</td></tr>
<tr><td>Tramadol</td></tr>
<tr><td rowspan="2">Injectable drugs/ biologics</td><td>Anti-NGFmAb (Added to evaluation list post initial meeting)</td><td>Subcutaneous</td></tr>
<tr><td>Pentosan polysulphate</td><td>I.M.</td></tr>
</table>

	Polysulphated glycosaminoglycan	I.M.
	Botulinum toxin	I.A.
	Corticosteroids	I.A.
	Hyaluronic Acid (low molecular weight)	I.A.
	Hyaluronic Acid (high molecular weight)	I.A.
	Platelet Rich Plasma	I.A.
	Stem cells	I.A.
	Opioids	I.M.
	Opioids	I.V. CRI
	Ketamine	I.V. infusions
	Lidocaine	I.V. infusions
Surgery	Preventive	
	Improve quality of life	

Appendix 2: Factors for consideration when selecting an NSAID or a canine anti-NGF mAb for dogs with osteoarthritis.

The availability, in certain geographies, of two different major classes of therapeutic (NSAIDs and the anti-NGF mAb) to control pain in dogs with clinical signs of OA is a relatively recent change in veterinary medicine. The differing modes of action and contrasting product attributes usefully expand the clinician's therapeutic toolbox, but the advantage of choice can also lead to uncertainty about when and how to incorporate each option most effectively into canine OA management protocols. Both classes are considered first-line options for the management of OA-associated pain and disability. Based on current registration state and available efficacy data, it is impossible, and inappropriate, to recommend one approach above the other. The efficacy study outcomes for NSAIDs and the anti-NGF mAb in dogs are very similar, and the treatments were evaluated in comparable populations of dogs. [1] [2] [3] [4] [5] [6] [7] [8] [9] That said, the COAST group members considered both to be appropriate first-line options for COAST stage 3 and 4 dogs, but for COAST stage 2 dogs, 3 of the COAST members indicated they would consider NSAIDs first in these dogs, while 6 COAST members indicated they would consider either an NSAID or anti-NGF as appropriate first-line options for COAST stage 2 dogs.

The potential benefit of concurrent use of these two therapeutic approaches is of veterinary interest. A two-week duration laboratory study evaluating the safety of concomitant administration of the canine anti-NGF mAb and an NSAID to healthy dogs without OA [10] reported no adverse events, including in joint pathology. This provides useful risk: benefit information for dogs requiring short-term administration of both products, but it is currently unknown if an NSAID and the anti NGF mAb can be used safely together for the long-term management of dogs with OA. Product development generally focuses on generating evidence of a benefit of use of the product when used alone. In phase 3 clinical studies for human medicine (human anti-NGF mAbs), the long-term combined use of an human anti-NGF mAb with an NSAID increased the risk of developing rapidly progressive osteoarthritis (RPOA) relative to the use of the human anti-NGF mAb alone. [11] Rapidly progressive osteoarthritis (RPOA) has not been described or recognized in dogs to date.

With the first anti-NGF mAb therapy being only recently approved, and relatively limited published reports on efficacy and safety of anti-NGF mAb therapy in general [10] [9] [12] [13], there is still a lot to be understood about this therapeutic approach in dogs and how to make treatment decisions regarding NSAIDs and anti-NGF therapies within OA management protocols. Until more data is available, the COAST Development Group believes that treatment choice should be guided by careful consideration of the following:

- Key pathophysiological processes that need to be controlled
- Product mode of action
- Product safety profiles
- Patient factors and requirements
- Product attributes

- Pet caregiver needs and preferences

For example, pain control is likely to be the primary consideration in all dogs with clinical signs of OA pain (COASTeR stages 2 to 4), supporting the use of either an NSAID or the anti-NGF mAb due to their demonstrated effectiveness for that purpose. Mode of action considerations may influence product choice, but currently there is no easy way to determine what pathological or neurobiological processes are driving pain in an individual patient. Inflammation may be a more significant contributor to OA pain in the earlier stages of the disease, whereas ongoing sensitization processes are thought to drive heightened levels of pain in more severely affected dogs. [14] Factors such as flexibility in dosing, complex medical comorbidities, convenience and compliance are all important drivers of product choice in dogs with OA-associated pain.

Safety considerations are important and each class tends to have its own safety profile characteristics. Package inserts detail safety data and product information in line with local approvals, and provide guidance on dosing, minimum age and weight recommendations and other considerations including co-morbidities and concurrent medications. Local approval guidelines should be followed for all products.

There are multiple needs for each patient, and it may be difficult to address all requirements. This places the emphasis on the veterinarian to identify the predominant requirements for that patient and select the treatment option that most closely addresses those prioritized needs.

References

[1] L. Rausch-Derra, M. Huebner, J. Wofford and L. A. Rhodes, "Prospective, Randomized, Masked, Placebo-Controlled Multisite Clinical Study of Grapiprant, an EP 4 Prostaglandin Receptor Antagonist (PRA), in Dogs with Osteoarthritis," *Journal of Veterinary Internal Medicine,* vol. 30, no. 3, pp. 756-763, 2016.

[2] A. Autefage, F. M. Palissier, E. Asimus and C. Pepin-Richard, "Long term efficacy and safety of firocoxib in the treatment of dogs with osteoarthritis," *Veterinary Record,* vol. 168, no. 23, p. 617, 2011.

[3] J. Murrell, E. Grandemange, F. Woehrle, J. Menard and K. White, "Clinical efficacy and tolerability of cimicoxib in dogs with osteoarthritis: a multicentre prospective study," *Open Journal of Veterinary Medicine,* vol. 2014, no. May, 2014.

[4] N. Reymond, C. Speranza, P. Gruet, W. Seewald and J. N. King, "Robenacoxib vs. carprofen for the treatment of canine osteoarthritis; a randomized, noninferiority clinical trial," *Journal of Veterinary Pharmacology and Therapeutics,* vol. 35, no. 2, pp. 175-183, 2012.

[5] P. B. Vasseur, A. L. Johnson, S. C. Budsberg, J. D. Lincoln, J. P. Toombs, J. G. Whitehair and E. L. Lentz, "Randomized, controlled trial of the efficacy of carprofen, a nonsteroidal anti-inflammatory drug, in the treatment of osteoarthritis in dogs," *Journal of the American Veterinary Medical Association,* vol. 206, no. 6, pp. 807-811, 1995.

[6] P. A. Doig, K. A. Purbrick, J. E. Hare and D. B. McKeown, "Clinical efficacy and tolerance of meloxicam in dogs with chronic osteoarthritis," *The Canadian Veterinary Journal,* vol. 41, no. 4, p. 296, 2000.

[7] M. B. Walton, E. C. Cowderoy, B. Wustefeld-Janssens, B. D. Lascelles and J. F. Innes, "Mavacoxib and meloxicam for canine osteoarthritis: a randomised clinical comparator trial," *Veterinary Record,* vol. 175, no. 11, p. 280, 2014.

[8] M. Salichs, L. Badiella, P. Sarasola and J. Homedes, "Efficacy and safety of enflicoxib for treatment of canine osteoarthritis: A 6-week randomised, controlled, blind, multicentre clinical trial," *Veterinary Record,* no. Sep, p. e949, 2021.

[9] M. J. Corral, H. Moyaert, T. Fernandes, M. Escalada, J. K. Tena, R. R. Walters and M. R. Stegemann, "A prospective, randomized, blinded, placebo-controlled multisite clinical study of bedinvetmab, a canine monoclonal antibody targeting nerve growth factor, in dogs with osteoarthritis," *Veterinary Anaesthesia and Analgesia,* vol. 48, no. 6, pp. 943-955, 2021.

[10] M. Krautmann, R. Walters, P. Cole, J. Tena, L. M. Bergeron, J. Messamore, D. Mwangi, S. Rai, P. Dominowski, K. Saad and Y. Zhu, "Laboratory safety evaluation of bedinvetmab, a canine anti-nerve growth factor monoclonal antibody in dogs," *The Veterinary Journal,* vol. 276, p. 105733, 2021.

[11] B. L. Wise, M. F. Seidel and N. E. Lane, "The evolution of nerve growth factor inhibition in clinical medicine," *Nature Reviews Rheumatology,* vol. 17, no. 1, pp. 34-46, 2021.

[12] B. D. Lascelles, D. Knazovicky, B. Case, M. Freire, J. F. Innes, A. C. Drew and D. P. Gearing, "A canine-specific anti-nerve growth factor antibody alleviates pain and improves mobility and function in dogs with degenerative joint disease-associated pain," *BMC Veterinary Research,* vol. 11, no. 101, pp. 1-12, 2015.

[13] R. P. Webster, G. I. Anderson and D. P. Gearing, "Canine brief pain inventory scores for dogs with osteoarthritis before and after administration of a monoclonal antibody against nerve growth factor," *American Journal of Veterinary Research,* vol. 75, no. 6, pp. 532-535, 2014.

[14] B. D. Lascelles, J. S. Gaynor, E. S. Smith, S. C. Roe, D. J. Marcellin-Little, G. Davidson, E. Boland and J. Carr, "Amantadine in a multimodal analgesic regimen for alleviation of refractory osteoarthritis pain in dogs," *Journal of Veterinary Internal Medicine,* vol. 22, no. 1, pp. 53-9, 2008.
